# Exploration of the optimal regimen of gastric mucosal cleansing medication for the *H. pylori* population before ME-NBI screening: study protocol for a single-center, single-blind, randomized controlled trial

**DOI:** 10.3389/fmed.2025.1516271

**Published:** 2025-04-02

**Authors:** Xinyi Mu, Yi Fan, Jingyu Xu, Rui Xie

**Affiliations:** ^1^Department of Nursing, Affiliated Hospital of Zunyi Medical University, Zunyi, Guizhou, China; ^2^Department of Endoscopy, Affiliated Hospital of Zunyi Medical University, Zunyi, Guizhou, China; ^3^Nursing College, Zunyi Medical University, Zunyi, Guizhou, China; ^4^Department of Endoscopy and Digestive System, Guizhou Provincial People's Hospital, Guiyang, Guizhou, China

**Keywords:** magnifying endoscopy with narrow-band imaging, *H. pylori*, early gastric cancer, randomized controlled trial, gastric mucosal cleanliness

## Abstract

**Objective:**

Magnifying endoscopy combined with narrow-band imaging endoscopy is an emerging method for early gastric cancer screening and diagnosis However, its effectiveness is closely related to the cleaning quality of the gastric mucosal preparation. *H. pylori* infection is a major risk factor for inadequate gastric mucosa cleaning quality preparation. Multiple medications are useful in helping patients with gastric mucosal cleansing preparations. This randomized controlled trial study protocol aims to investigate the effect of different combinations of medications on the quality of gastric mucosal cleansing in an *H. pylori*-infected population.

**Methods:**

This study is a prospective, randomized, single-blind, single-center trial. The subjects are patients who require magnifying endoscopy combined with narrow-band imaging and have evidence of *H. pylori* infection (a non-invasive diagnostic ^13^C urea breath test was used to examine the study subjects). These patients will be randomly assigned to the control group (Group A) and the experimental groups (Groups B, C, D, E, and F). Each group will consist of 44 patients, with a total of 264 patients expected to be enrolled. The core content of the drug preparation regimen for each group is as follows: Group A (control group) will take 10 ml of simethicone before the examination; Group B (experimental group) will take 20,000 units of pronase before the examination; Group C (experimental group) will take 600 mg of N-acetylcysteine before the examination; Group D (experimental group) will take 10 ml of simethicone +20,000 units of pronase before the examination; Group E (experimental group) will take 10 ml of simethicone + 600 mg of N-acetylcysteine before the examination; Group F (experimental group) will take 10 ml of simethicone + 20,000 units of pronase + 1 g of sodium bicarbonate before the examination. All group medications will be dissolved in 50 ml of warm water at 20–40°C. All patients will fast for ≥6 h and abstain from drinking for 2 h before the examination. The primary endpoint is the gastric mucosa cleanliness score. Secondary endpoints include the early detection rate of gastric cancer, polyp detection rate, adenoma detection rate, procedure time, number of irrigations, patient medication compliance, preoperative anxiety, incidence of adverse reactions, overall patient satisfaction, and willingness to undergo the examination again.

**Implications:**

The results of this research project are aimed at improving the quality of gastric mucosal cleansing preparations in the *H. pylori* population to meet the demand for early diagnosis and treatment prevention screening for early gastric cancer screening. The implementation of the results of the study and their inclusion in the guidelines may reduce economic expenditures by reflecting a reduced need for social and health care services.

**Clinical Trial registration:**

Chinese Clinical Trial Registry (ChiCTR). Number of identification: (ChiCTR2400087510).

## 1 Introduction

In the twenty-first century, cancer remains a major social, public health and economic problem worldwide. According to the latest report of the International Agency for Research on Cancer (IARC), gastric cancer accounts for 4.9% of cancer incidence and 6.8% of cancer deaths, ranking fifth globally in terms of incidence and mortality (1). As we all know, *H. pylori* infection is closely related to digestive diseases such as chronic gastritis, peptic ulcer, gastric cancer and mucosa-associated lymphoid tissue lymphoma. The World Health Organization has classified *H. pylori* as a group I carcinogen for human gastric cancer. Studies have shown that the global *H. pylori* infection rate is 48.5%, and the *H. pylori* infection rate in China is as high as 50% (2). *H. pylori* is considered to be the most important part of the risk factors associated with GC, and it has been estimated that more than 78% of the population with GC is directly related to *H. pylori* infection (3). Therefore, selection of patients with suspected *H. pylori* infection because they are at a higher risk of gastric cancer compared to the general population, and it is also important for accurate gastric cancer risk stratification.

The survival period of gastric cancer patients is closely related to the time of clinical diagnosis (early stage can be cured, while late stage can only be treated with palliative symptomatic therapy, etc.). According to the developmental pattern of gastric cancer, the whole course of the disease usually ranges from 5 to 15 years, which provides a valuable window of time for early clinical screening and subsequent treatment. However, the key problem is that early gastric cancer symptoms are not obvious or even asymptomatic. Most of the patients are already in the middle or late stage of gastric cancer when they visit the doctor, and early diagnosis and treatment is the key to early gastric cancer. Some studies have shown that the 5-year survival rate of gastric cancer can reach more than 90% after high-quality upper gastrointestinal endoscopy screening and treatment (4–7).

Plain white light endoscopy is inadequate for the diagnosis of early precancerous lesions and EGC. Especially in the *H. pylori* population Narrowband imaged magnifying endoscope (ME-NBI) is a combination of magnifying endoscope and narrowband imaging, due to its unique optical method, fine mucosal structures and microvessels can be observed using an 80 × magnifying endoscope. It is specifically designed for the detection of early gastric cancer (8).

A clear visualization of the gastric mucosa is critical to prevent the oversight or misdiagnosis of early-stage gastric cancer lesions (9, 10). During gastrointestinal endoscopy, the quality of the examination can be significantly compromised by secretions on the mucosal surface, including saliva, bubbles, and mucus. Inadequate preparation of the gastric mucosa not only impairs visual clarity but also heightens the risk of overlooking small lesions and decreases the detection rate of upper gastrointestinal tumors. Conventional cleaning methods, such as repeated suction or saline irrigation during the procedure, not only extend the duration of the operation but also exacerbate patient discomfort and increase the risk of intraoperative complications. Therefore, ensuring thorough preparation for gastric mucosa cleanliness is essential for enhancing both the efficiency of endoscopy and diagnostic accuracy.

To achieve optimal visualization of the gastric mucosa, it is essential to administer preoperative medications aimed at eliminating mucus and foam within the stomach (11). Due to variations in clinical practices across different medical institutions, there is currently no consensus among major endoscopy centers regarding the necessity and selection criteria for preoperative medication regimens for gastric mucosa preparation. However, studies have confirmed that combination therapy yields superior results (12, 13). Research into enhancing the quality of gastroscopy has a long history. American scholar J. Alfred Rider first proposed the use of antifoaming agents to improve the visibility of the gastric mucosa in the 1960s (14). Simethicone is a non-ionic surfactant that effectively reduces the surface tension of liquids, thereby facilitating the rupture and expulsion of gas bubbles within the stomach. This action significantly enhances the visual field during endoscopic procedures. Its application in gastroscopy was first documented in 1954 (15), and it has since become widely utilized in preoperative preparations to improve the detection rate of minor lesions (16). Pronase is a glycoprotein hydrolase that effectively degrades the mucus layer on the surface of the gastric mucosa, thereby enhancing endoscopic visibility (17). Research has demonstrated that this agent can also diminish gastric wall echogenicity during endoscopic ultrasound examinations, leading to improved image quality (18). However, due to the high acidity present in the stomach, Pronase's activity may be constrained; thus, its use in conjunction with sodium bicarbonate or scopolamine butylbromide is recommended to optimize its functional environment. Furthermore, existing clinical studies indicate that Pronase can enhance visual clarity within the gastric fundus and body, significantly increasing the detection rate of small lesions such as erosions and ulcers (19). N-acetylcysteine has the ability to disrupt disulfide bonds in mucus, thereby reducing its viscosity and improving mucosal visualization during gastroscopy (20). Research indicates that the combination of this medication with simethicone significantly enhances the imaging quality of gastroscopy while decreasing the need for irrigation fluid (16, 21). Moreover, when compared to traditional mucolytic agents such as chymotrypsin and pronase, N-acetylcysteine may demonstrate superior efficacy; however, further studies are required to establish its safety across diverse populations.

Current research predominantly concentrates on pre-endoscopic medication regimens for the general population. In contrast, studies addressing optimal medication protocols for high-risk groups susceptible to gastric cancer, such as patients with *H. pylori* infection, remain relatively limited. This limitation affects the accuracy of our screening for high-risk groups of early gastric cancer, hence, there is a need to conduct a study to address this clinical issue.

## 2 Objective

This study employs a single-center randomized controlled trial design, aiming to investigate whether the combined use of multiple defoaming agents and mucolytic agents, including simethicone, pronase, N-acetylcysteine, and sodium bicarbonate, prior to magnifying endoscopy with narrow-band imaging, can significantly enhance the clarity of the gastric mucosa in patients infected with *H. pylori*. Additionally, we have incorporated several observational metrics into the protocol to assess the safety of the procedure and the effectiveness of reducing the operation time.

## 3 Materials and methods

### 3.1 Study design

**Figure 1:**
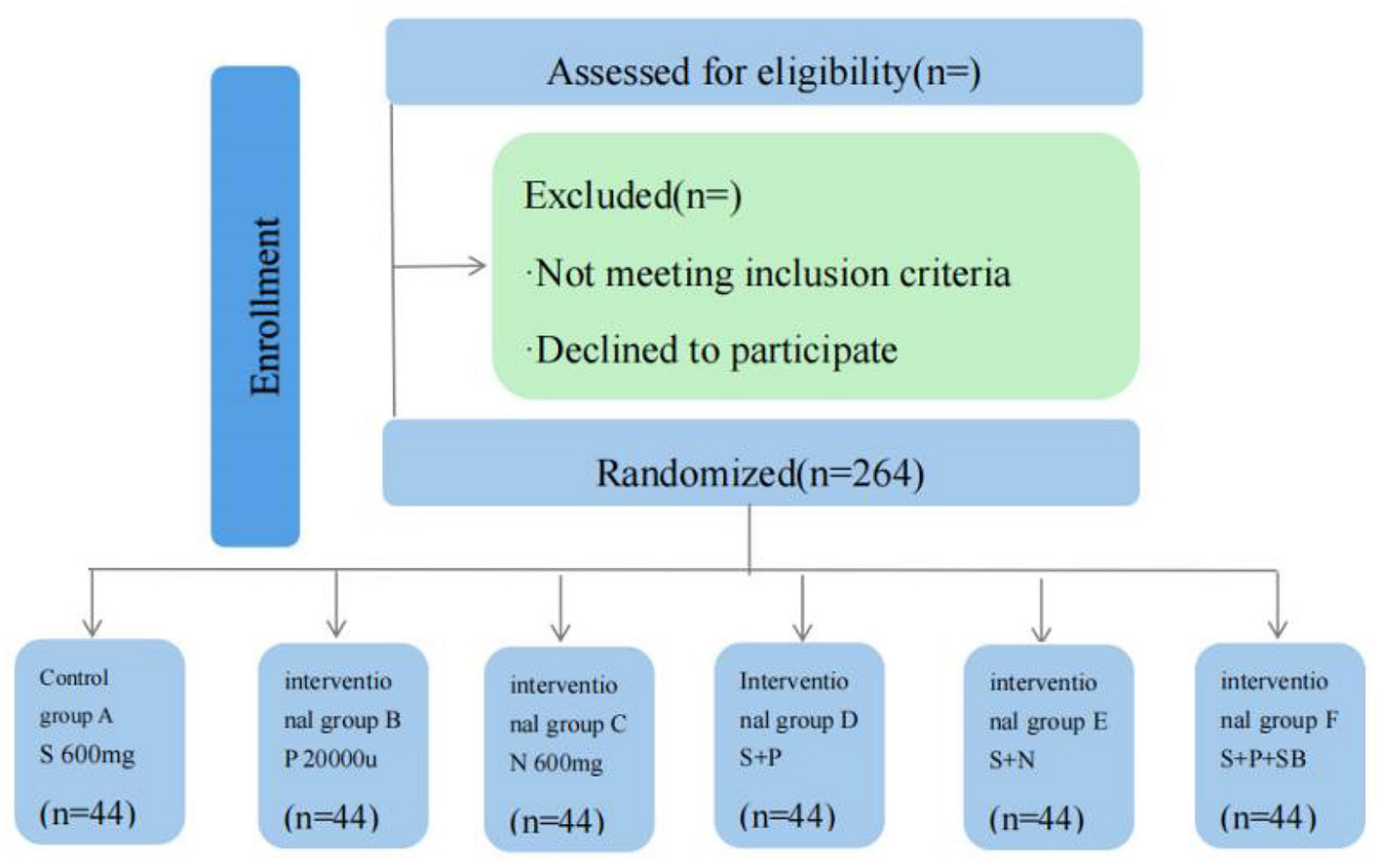
Flowchart of the patient enrolment procedure.

The study is a prospective, single-center, single-blind, randomized controlled study. It is planned to be conducted from September to December 2024 at the Center for Gastrointestinal Endoscopy, Affiliated Hospital of Zunyi Medical University, Zunyi, Guizhou Province, China. The endoscopists participating in this study are experienced endoscopists who perform more than 800 magnified gastroscopy narrow-band imaging techniques per year. The study protocol was approved by the Biomedical Research Ethics Committee of the Affiliated Hospital of Zunyi Medical University and registered. The patient enrollment flowchart is shown in Figure 1.

### 3.2 Blinding

Due to the nature of the intervention, all participants and endoscopists involved in the study were blinded to ensure the authenticity and objectivity of the data. The endoscopic procedures were performed by a single endoscopist with over 1000 ME-NBI procedures per year, and the quality of acquired images was analyzed and evaluated by two gastroscopy experts with ≥10 years of experience (with more than 500 cases reviewed). Initial baseline data collection will commence, with two other well-trained researchers (blinded to the groups) responsible for data collection. The investigator responsible for implementing the intervention will not be involved in the data collection process.

### 3.3 Participants and recruitment

#### 3.3.1 Eligibility criteria

##### 3.3.1.1 Inclusion criteria

- Age ≥18 years and ≤65 years male or female (22);
- According to the diagnostic criteria for *H. pylori* infection (23), a non-invasive diagnostic ^13^C urea breath test was used to examine the study subjects. Patients were instructed to avoid using antibiotics, bismuth preparations, or proton pump inhibitors for 2 weeks prior to the test. The labeled urea solution was administered: typically, a ^13^C-labeled urea solution at a dose of 75 mg/m^2^ of body surface area. Breath samples were collected: baseline breath samples were taken to measure the baseline values. After consuming the labeled urea, breath samples were collected at fixed intervals (10 min). The abundance of ^13^C in the breath samples was measured using mass spectrometry. The results were expressed as δ values (i.e., the ratio of ^13^C/^12^C), with a threshold of 3.5‰; a δ value above this threshold was considered positive. A positive result: δ value > 3.5‰, indicating *H. pylori* infection. A negative result: δ value ≤ 3.5‰, indicating no *H. pylori* infection.
- No previous magnified gastroscopy with narrow-band imaging;
- Ability to sign an informed consent form on their own.

##### 3.3.1.2 Exclusion criteria

- A confirmed diagnosis or high suspicion of gastric cancer or other malignant tumors of the gastrointestinal tract;
- Have a history of gastric surgery or other surgeries affecting gastric function;
- Having serious heart, liver, kidney, and other organ insufficiency or other serious systemic diseases;
- Having factors affecting the clarity of the gastric mucosa, such as heavy smoking, alcohol consumption, and taking non-steroidal anti-inflammatory drugs (24– 26);
- Having received *H. pylori* eradication therapy or other antimicrobial therapy within the past month;
- Pregnant or lactating women.
- Allergy to any of the study medications.

#### 3.3.2 Sample size

The gastric mucosa clarity score was used as the primary observation indicator, and the sample size calculation formula for multiple group means was evaluated using PASS software. The significance level (α) was set at 0.05 and β at 0.1. The estimated minimum sample size required for each group was 40 cases, totaling 240 cases, with an expected dropout rate of 10%. Both the control and experimental groups had 44 cases each. The calculation formula is as follows:


$$n=\frac{\varphi^{2}\left(\sum_{j=1}^{k}s_{j}^{2}/k\right)}{\sum_{j=1}^{k}{\left(X_{j}-X\right)}^{2}/\left(k-1\right)}.$$

#### 3.3.3 Recruitment

The recruitment exercise was carried out between September and December 2024, and a total of 264 participants were recruited to administer outpatient screening at the Digestive Endoscopy Center. To effectively screen eligible participants, study team members will work with outpatient staff to review medical records for patients with high blood pressure, diabetes, or dyslipidemia. Patients who meet the eligibility criteria will be contacted by one of the researchers to gauge their interest in learning more about the research project. Participants will then be contacted by another skilled researcher at the Endoscopy Center via investigator phone over 2 days to provide detailed information about the study. Qualified subjects will be recruited after a signed informed consent form is obtained.

### 3.4 Randomization and allocation concealment

In this study, a table of 264 random numbers of cases was randomly generated by one endoscopy staff member using the computer SPSS and stored in a sealed envelope by another endoscopy staff member in the order in which the patients were scheduled for examination. When a patient who meets the inclusion criteria agrees to participate in this study, we will open the envelopes in order and randomize the patients into 6 subgroups A.B.C.D.E.F according to the numbers shown in the envelopes, with a randomization ratio of 1:1:1:1:1:1. Two staff members were not involved in this study. None of the endoscopic operators who performed the examination know which subgroup medication the patient was taking, the purpose of which was to ensure the truthfulness and objectivity of the data. Endoscopy operations were performed by the same endoscopist with more than 1,000 ME-NBI operations/year, and the quality of the acquired images was analyzed and evaluated by two gastroscopy reading specialists with ≥10 years of experience (>500 readings).

### 3.5 The intervention procedure

All enrolled patients are required to consume a light and easily digestible diet 1 day before the examination and begin fasting 6 h prior to the examination, with no liquids allowed 2 h before the procedure (27). For the participants of this study and their families, we will provide comprehensive information regarding the informed consent process. One hour before the commencement of the trial, staff will accurately fill in each patient's number on the top right corner of the basic information questionnaire based on the number of patients scheduled for screening that day, and strictly prepare the gastric formulation according to the specified drug ratio. Additionally, according to the prepared blind protocol, patients will be assigned to the corresponding groups based on their different numbers, and medication will be distributed 30 min before the examination (28). In control group A, the patients consumed only 50 ml of simethicone 600 mg in water, in experimental group B, the patients consumed 50 ml of pronase 20,000 u in water, in experimental group C, the patients consumed 50 ml of N-acetylcysteine 600 mg in water, and in experimental group D, the patients consumed 50 ml of simethicone 600 mg and 20,000 u of pronase mixed in water, and in experimental group E, the patients consumed 50 ml of simethicone 600 mg and N-acetylcysteine 600 mg. In experimental group F, patients drank 50 ml of simethicone dissolved in water 600 mg + 20,000 u pronase and 1 g of sodium bicarbonate solution.

After drinking the medication, patients will be professionally instructed by nurses “1–1” to perform position reversal training, including right side position, prone position, left side position, and flat position, and each position should be uniformly breathed for 10 times, and 5 consecutive position changes should be performed (29). In addition, we placed MP3 audio and animated videos in the examination waiting room, in the form of a Mandarin version and a dialect version (to accommodate patients with different literacy levels), with a duration of 4 min, which were used to instruct the patients in the correct operation of turning. Throughout the process, nurses will follow the instructions to ensure that the patient has a clear view of the mucosal background so that the physician can perform an accurate examination. Meanwhile, all patients will receive deep sedation with propofol at 1.5–2.5 mg/kg in the presence of a qualified anesthesiologist, administered at 5–7 min intervals. If the depth of sedation is insufficient, 0.5 mg/kg of propofol will be slowly pushed until proper sedation is achieved. During deep sedation, we will closely monitor the patient's vital signs, including pulse, oxygen saturation, heart rate, blood pressure, and other key indicators. In addition, all patients will receive 4–5 L/min of supplemental oxygen through an oxygen mask to ensure their comfort and safety during the examination.

During the examination, the patient was placed in the left lateral position with the head slightly tilted forward and the legs flexed. After the anesthesiologist confirmed that the patient had reached a state of deep sedation, the same senior endoscopist inserted the endoscope through the mouth. Under direct endoscopic visualization, the procedure began at the upper esophagus, sequentially examining the upper esophagus, lower esophagus, cardia, upper gastric body, lower gastric body, gastric antrum, gastric angle, and gastric fundus. Special emphasis was placed on performing narrow-band imaging (NBI) from the hypopharynx to observe the hypopharynx and esophagus, and 20 magnified gastroscopy and NBI images were captured separately (30). A researcher with a master's degree in nursing recorded the patient's vital signs and observational indicators during the examination. At the end of the examination, the image data obtained from the subjects were scored for gastric clarity by two experienced endoscopists (the reading physicians were unaware of the preoperative gastric preparation regimen the patients had received in this study). They also recorded the presence of any suspicious lesions and the pathological results of subsequent routine gastroscopy reviews. In case of disagreement in the assessment, a third endoscopist made the final decision. After the patient regained consciousness from anesthesia, they were asked to complete a feedback questionnaire regarding their satisfaction with the ME-NBI examination and were informed of post-examination precautions. The nursing researcher followed up with the patient via telephone or other mobile means the next day to inquire about any adverse events they might have experienced.

### 3.6 Outcomes and measurements

Patients participating in the trial underwent a baseline assessment conducted by a Master of Nursing researcher who was blinded to the group assignments. The baseline assessment data will encompass, but not be limited to, demographic factors such as age, gender, marital status, level of education, employment status, socioeconomic status, dietary habits, severity of illness, medical history, mental state and sleep patterns.

#### 3.6.1 Primary outcomes

The primary objective of the study is to compare the effects of different preoperative medication preparation regimens on the cleanliness of the gastric mucosa in individuals infected with Helicobacter pylori. To achieve this goal, we referred to the scoring criteria proposed by Kuo et al. (31), using the gastric mucosa cleanliness score as the primary outcome measure. The visibility of four parts of the stomach—namely the antrum, the lower body, the upper body, and the fundus—was scored separately. The scoring system ranges from 1 to 4 points, and the total score for each patient is calculated based on the visibility of their gastric mucosa. Two additional senior endoscopists independently scored the images of each patient. In cases of scoring discrepancies, a final consensus score was determined through joint consultation. The clarity of each image was assessed according to specific criteria. The classification criteria for gastric mucosal cleanliness are shown in Table 1.

**Table 1:** Gastric mucosal clarity scale.

**Scale**	**Content**
Score 1	No adherent mucus and clear views;
Score 2	A thin coating of mucus that does not obscure views
Score 3	Gastric antrum, stomach body, fundus a little foam, blurred vision, need to wash with a small amount of water (< 30 ml);
Score 4	Almost all gastric mucosa has very thick mucus, blurred vision, need to wash with more water (>30 ml).

#### 3.6.2 Secondary outcomes

We also hypothesized that enhancing the clarity of the endoscopic field of view would facilitate early detection of gastric cancer in patients, increase patients' interest in early cancer screening, and enhance their understanding of amplification endoscopic narrowband imaging. This may ultimately lead to a higher detection rate of abnormal lesions, reduced anxiety, and improved patient experience. Therefore, we have established the following secondary endpoints. Secondary outcomes are shown in Table 2.

**Table 2:** Secondary endpoint indicators.

**Item**	**Content**
Detection rate of early gastric cancer	Detection rate of early gastric cancer = number of pathologically confirmed cases/total number of endoscopy cases × 100%.
Polyp detection rate	Record the number, location and nature of polyps found during gastroscopy, and perform biopsy or excision for pathological examination.
Adverse reactions to taking the experimental drug	The patients' discomfort reactions, such as nausea, vomiting, abdominal pain, bloating, throat discomfort, etc. from oral drug solution to the end of the gastroscopy were recorded, and their frequency and severity were measured.
Gastric mucosal irrigation times	In the process of gastroscopy, when there is foamy mucous in the gastric mucosa of the patient, to observe and clear the gastric mucosa, the gastric mucosa should be rinsed with distilled water for about 30 ml each time.
Endoscopic time	Refers to the time required for the gastroscope to enter the oral piriform fossa at the entrance of the esophagus to the exit of the gastroscope at the end of the examination, in seconds (s), and excludes the time for biopsy.
Microscopic lesion detection rate	The number, location, and nature of lesions < 5 mm in diameter found during gastroscopy were recorded and biopsied or excised for pathological examination.
Willingness to re-examine	Willingness to check again if needed.
Preoperative anxiety	Preoperative anxiety was assessed using a self-rating scale of 0 to 10 points (measured by self-rated sleep quality before surgery [where 0 indicates very poor sleep quality and 10 indicates the same sleep quality as usual].

### 3.7 Data collection

During the appointment and on the day of the gastroscopy, data will be collected using a standardized case report form.

### 3.8 Data analysis

We will use spss29.0 software for statistical analysis of data. Descriptive statistical analysis will be used for general baseline assessment data (demographic factors such as age, sex, marital status, education level, employment status, socioeconomic status, dietary habits, severity of illness, medical history, mental state, sleep, etc.). The Chi-square test will be used to compare the distribution of categorical baseline variables across the five intervention groups. Differences in continuous variables will be analyzed using one-way analysis of variance (ANOVA) or non-parametric rank sum tests. Six groups of gastric mucosa used bubble condition level rank and inspection were analyzed, and the detection rate of early gastric cancer and polyp is used chi-square test, operation time and clean degree of gastric mucosa To Mean +/– SD, according to the single factor analysis of variance was used to test, by using the least significant difference method compare two.

#### 3.8.1 Missing data plan

Although our trial recruitment was performed strictly according to standards, data loss was inevitable when subjects dropped out during the examination for reasons beyond our control. The researchers will comprehensively and systematically check the questions of the subjects from enrollment to the end of the examination to determine the situation that the subjects did not fill out and send reminders in time. In addition, we set up an outcome assessment in the field, where researchers will review questionnaires and remind participants to fill in missing data immediately. Assuming that the missing data is completely random, the reasons for non-compliance and non-retention will be recorded.

#### 3.8.2 Data quality control

To ensure the integrity and reliability of data collection, all researchers at research institutions receive standardized training on research protocols and data collection quality control techniques before the study. Access to and use of anonymized data for all subjects will be limited to authorized members of the research team. In addition, subjects receiving different subgroup drug interventions may discuss these. To minimize data contamination, subjects will be instructed not to send, share, or exchange this information with others until the end of the study.

## 4 Discussion

To the best of our knowledge, this study will be the first to explore the effect of different gastric mucosal preparation protocols on the clarity of gastric mucosa in gastric cancer risk groups in endoscopy, and the success of this randomized controlled trial will help the clinical staff to establish a more complete set of gastric mucosal preparation protocols to improve the early detection rate of gastric cancer in gastric cancer risk groups. According to the Kyoto Global Consensus Report on Gastritis (32), the self-cleaning ability of the gastric mucosa is weakened in *H. pylori*—positive patients, and the surface of the gastric body tends to be covered with more whitish and turbid mucus, so that more turbid mucus is retained in the stomachs of *H. pylori*—positive patients compared with that of healthy individuals. As a result, conventional preparation protocols are unable to optimize their preoperative preparation, and the existing means of repeated rinsing during gastroscopy, although it can solve this problem to a certain extent, has the problems of increasing the length of the examination and aggravating the patient's postoperative reactions, etc. Therefore, we hope that this study will find a useful preparation protocol through scientific exploration to improve clarity in the endoscopic field of view and to reduce the patient's Preoperative anxiety and adverse reactions.

Based on evidence-based evidence, this study designed a multi-group experimental protocol under the same experimental conditions for different drugs (33–37)—all of which are relatively effective in improving gastric mucosal clarity in previous experiments. We expect to use this experimental design to identify protocols to improve mucosal clarity and thus detection rates during endoscopy. We sincerely hope that this experiment will achieve such a goal.

Of course, there are some shortcomings in this study, firstly, it is a single-center randomized controlled study, and although the experimental data from this study may indicate whether the group protocol is effective in improving gastric mucosal clarity, further validation of the protocol in multiple institutions could be considered in the future. In addition, the identification of mucosal clarity in *H. pylori*-positive patients is potentially affected by factors such as the examiner's technique, duration of medication, active position, and the patient's own body; therefore, we used standardized training and the same equipment to minimize these potential effects, and future researchers can build on our study to explore more factors.
